# Hydrogen-rich water mitigates zinc oxide nanoparticle toxicity in arugula: a focus on growth and antioxidant defense

**DOI:** 10.1186/s12870-026-09526-2

**Published:** 2026-07-20

**Authors:** Huwida A. A. Abdel-Kader, Afaf M. Hamada, Fatma A. Farghaly

**Affiliations:** Botany and Microbiology Department, Faculty of Science, Assiut University, Assiut, 71516 Egypt

**Keywords:** Enzymatic antioxidant, *Eruca sativa*, Non-enzymatic antioxidant, Oxidative stress

## Abstract

The escalating use of zinc oxide nanoparticles (ZnO-NPs) necessitates a deeper understanding of their phytotoxicity in edible plants. Although hydrogen-rich water (HRW) mitigates abiotic stress, its capacity to counteract ZnO-NP damage in arugula (*Eruca sativa* L.) remains unexplored. The capacity of exogenous HRW at 50% and 100% saturation to mitigate ZnO-NP (150 mg L^− 1^) toxicity in arugula was investigated. Four treatment groups were established: control, ZnO-NPs alone, and ZnO-NPs combined with 50% or 100% HRW. Physiological and biochemical responses were evaluated in both seedlings and mature leaves. HRW effectively counteracted ZnO-NP stress and enhanced growth, increasing the tolerance index (TI) by 12.37% (50% HRW) and 17.74% (100% HRW) in early stages. HRW treatments at 50% and 100% increased shoot growth by 67.73% and 83.95% and root growth by 165.49% and 130.97%, respectively. Photosynthetic pigments were recovered by an average of 30.96% (50% HRW) and 43.78% (100% HRW). Furthermore, 100% HRW boosted superoxide dismutase (SOD) activity by 34.01%-89.85% and catalase (CAT) activity by 55.10%-29.78% in leaves and seedlings, respectively. Conversely, ZnO-NP exposure elevated zinc (Zn) concentrations and translocation factor (TF) and stimulated oxidative stress markers, including malondialdehyde (MDA), anthocyanins, ascorbic acid (AsA), proline, and peroxidase (POD); HRW application reversed these damaging effects. A stage-specific stress response was observed: seedlings accumulated higher concentrations of hydrogen peroxide (H₂O₂) scavengers, including cysteine (Cyst), glutathione (GSH), phenolics, and soluble proteins, whereas mature leaves showed decreases in these levels. HRW mitigated the accumulation of these protective compounds in seedlings while boosting them in leaves. These results suggest that HRW treatments have practical implications for enhancing arugula’s capacity to tolerate ZnO-NP exposure. This study offers crucial mechanistic insights and robust data for environmental toxicologists. Furthermore, it provides a valuable reference for the potential environmental applications of HRW in contaminated agricultural systems.

## Introduction

Hydrogen (H_2_) is among the most common gases on Earth. According to reports, H_2_ is a novel gaseous signal molecule with antioxidant properties that prevent oxidative damage and inhibit cytotoxicity [1, 2]. While direct H₂ use is limited by its hazardous and combustible nature [3], hydrogen-rich water (HRW) provides a safe, economical, and accessible medium for experimental studies. Applying HRW to various plants can protect and improve their performance against abiotic stressors, such as heavy metals [4]. The primary mechanism of HRW activity might be preferential reactive oxygen species (ROS) scavenging [2, 5]. Most studies indicate that HRW enhances antioxidant enzyme activities based on regulatory processes [6, 7]. Additionally, HRW maintained the redox balance by raising antioxidants [4, 6, 8, 9].

Zinc (Zn) is the second most prevalent transitional metal in living things [10]. Because of its activating, structural, and catalytic properties, Zn is essential for plant growth, reproduction, and signaling [11, 12]. Zn is toxic beyond the ideal range, but it is necessary for plant growth below that point. Due to their widespread application in the nano-industry, zinc-based nanoparticles (NPs) are produced in much larger quantities (550–5550 metric tons) than other NP kinds [13–15]. The potential ecological and toxicological consequences of zinc oxide nanoparticles (ZnO-NPs) for flora and fauna have raised significant societal concerns. The potential risk associated with ZnO-NPs is complex and multifaceted, depending on characteristics such as their size, surface charge, and shape, as well as environmental factors like ion release, soil vs. hydroponic culture, exposure concentration, atmospheric conditions (temperature, humidity, light), and the plant species involved.

The effects of ZnO-NPs largely depend on their concentration, potentially leading to either beneficial or detrimental outcomes [16, 17]. Increased environmental exposure to NPs raises concerns about their potential for detrimental interactions, either antagonistic or synergistic, with other ecological factors [18, 19]. The behavior and toxicity of binary or multiple NP complexes remain poorly understood, introducing an additional layer of complexity to the risks already associated with individual NPs. As ZnO-NPs’ particle size shrank, more oxygen (O_2_) gaps appeared on their surface. Under some circumstances, this can encourage electron-hole pairs to combine with hydroxyl (^•^OH) ions and O_2_ molecules to create reactive oxygen species (ROS) [13]. This process is widely known to produce ROS and cause oxidative stress, which can lead to cell damage due to ZnO-NPs [14]. Because ZnO-NPs occupy defense molecules against oxidative stress, they may indirectly generate ROS [20]. This event can result in increased quantities of hydrogen peroxide (H₂O₂) and ^•^OH, as well as notable decreases in mitochondrial membrane potential [13]. However, antioxidant systems were insufficient to restore the generated ROS from ZnO-NP exposure. Failure to remove ROS promptly disrupts the cellular balance, leading to oxidative damage [13, 21, 22].

As lifestyles become more sedentary and food habits change, there has been a surge in demand for, and consumption of, nutrient-dense natural foods. Packed with sulfur-rich compounds, coloring pigments, minerals, vitamins, dietary fiber, and other bioactive compounds, they possess a distinctive nutraceutical status and offer well-documented therapeutic benefits [23]. Some vegetables, like arugula (Brassica family), are popular dietary staples worldwide, but their cultivation in polluted soil raises potential health concerns. Due to their abundance of health-promoting substances such as vitamin C, carotenoids, flavonoids, glucosinolates, phenolic compounds, and fibers, arugula species are very desirable as green leafy vegetables [23, 24]. Leafy vegetables are vulnerable to environmental factors that affect the quality and quantity of their phytonutrient profiles before and after harvest [25]. Sturm and Wagner [26] reported that Brassicaceae vegetables, such as arugula, could offer health benefits by lowering the risk of chronic illnesses like cancer. This is because sulfur-containing plants, also known as glucosinolates, are present in Brassicaceae family veggies, which have anti-inflammatory and anti-cancer properties and are beneficial for health. Apart from food, arugula serves many other applications, including as a source of seeds and oil extraction. Arugula has a quick biological cycle (between 30 and 60 days). Also, it is known for its extensive spectrum of phytochemicals, making it not only traditionally useful but also economically valuable [27]. Although arugula is known to uptake heavy metals (HMs), ZnO-NP absorption has not been studied [27].

While HRW is known for shielding plants from diverse environmental stresses, its potential to counteract damage from ZnO-NPs in edible crops like arugula remains largely uninvestigated. Understanding the impact of ZnO-NPs is vital for food safety and sustainable agriculture. Our study fills a crucial gap by investigating their harmful effects on arugula, a common leafy green, and, more importantly, by exploring a novel and promising way to mitigate that damage. Addressing this research gap is crucial, as ZnO-NPs are ubiquitous, and HRW offers a promising, environmentally friendly mitigation strategy. Investigating this popular edible crop ensures the findings have direct relevance for food security and advancing sustainable agriculture. Our study provides novel, detailed mechanistic insights. Instead of just showing HRW’s protective effects, we comprehensively analyzed its physiological and biochemical impact. By assessing plant growth, photosynthetic pigments, and antioxidant defenses, the study clarifies how HRW mitigates ZnO-NP stress throughout early and late growth stages. To ensure the validity of our findings, the study incorporates rigorous statistical analysis.

## Materials and methods

### Chemicals

Chemicals used in this study were purchased from Sigma-Aldrich (St. Louis, MO, USA). Spectrophotometric readings were taken at 25 °C with a Unico UV-2100 spectrophotometer (United Products & Instruments Inc. (UNICO) New Jersey, USA).

### Preparation of hydrogen-rich water

Hydrogen-rich water (HRW) was prepared by saturating one liter of distilled water with high-purity (99.99% v/v) hydrogen gas. This was done using a hydrogen gas generator (SHC-300, Saikesaisi Hydrogen Energy Co., Ltd., Shandong, China) at a flow rate of 150 mL min^− 1^ for about an hour [8]. The saturated solution (100% HRW) that was produced had a pH of 6.32; it was then diluted with distilled water to produce 50% (v/v) HRW solutions. Gas chromatography (Shimadzu, GC-2014, Kyoto, Japan) confirmed the 100% HRW solution had a hydrogen concentration of 36,029 µM. A hydrogen concentration of roughly 18,0145 µM was observed in the 50% HRW. Both solutions maintained stable hydrogen concentrations for at least 12 h at 25 °C [8].

### Preparation of zinc oxide nanoparticles and characterization

ZnO-NPs were obtained from Sigma-Aldrich Company. These NPs were 99.5% pure, had a surface area of 15–25 m^2^ g^− 1^, and featured particle sizes less than 100 nm. ZnO-NPs were dispersed in distilled water via a horn tip sonicator (Bandelin Sonopuls HD 3200 Ultrasonic homogenizer, Germany) to produce a uniform suspension.

The Fourier Transform Infrared (FTIR) spectrum of the ZnO NPs was recorded from 4000 to 400 cm⁻¹ using the KBr pellet method. For TEM analysis (JEOL TEM 100 CXII, Electron Microscope Unit, Assiut University, Egypt), a droplet of the NP suspension was deposited onto carbon-coated copper grids and air-dried to evaluate particle size and morphology.

### Plant growth and experimental design conditions

Arugula seeds (*Eruca sativa* L.) were obtained from the Agricultural Research Center, Ministry of Agriculture, Giza, Egypt. The seeds were sterilized and soaked for eight hours at 25 °C with aeration in three solutions: distilled water (as a control), 50% HRW, and 100% HRW. Presoaked seeds were planted in Petri dishes and irrigated with either 6 mL of distilled water or 6 mL of 150 mg L^− 1^ ZnO-NPs. This specific ZnO-NP concentration was selected following preliminary tests and Farghaly et al. [28]. The seedlings’ emergence was observed periodically on the first, second, and third days following the various treatments. After the growth parameters were established, the seedlings were frozen at -80 °C to allow for additional analysis. To measure dry weight (DW), distinct seedling sets were dried at 60 °C. The tolerance index (TI) was determined [29]. The ratio was measured as follows:

$$\mathrm{TI}\%=\text{DW of the ZnO-NP-treated seedling/DW of the control}\times100$$

Seven-day-old seedlings were transplanted into plastic-lined pots filled with 2.5 kg of a 2:1 (v/v) soil-sand mixture and irrigated to 100% field capacity. Seven-day-old seedlings were examined in petri dishes, and only healthy seedlings were selected for transplantation. Each seedling was carefully removed with a sterile spatula; the root plug was supported from beneath to prevent root damage. To protect delicate stems, seedlings were handled only by the leaves or root plugs. Rapid transplantation minimized root exposure and desiccation, ensuring optimal seedling viability and establishment. A sterile dibbler was used to make holes in the potting mixture slightly larger than the root plugs for seedling transplantation. Each seedling was carefully inserted so that the original soil line remained level with the surface of the new growth medium. Special attention was given to maintaining direct contact between the root hairs and the surrounding substrate to promote a successful establishment. The potting mixture surrounding the seedling base was gently pressed to eliminate air pockets while avoiding excessive compaction that could restrict drainage and root aeration. The composition of the experimental soil included the following: 1.76% organic matter, 360 mg kg^-1^ Ca^2+^, 183 mg kg^-1^ HCO_3_^-^, 37.8 mg kg^-1^ K^+^, 144 mg kg^-1^ Mg^2+^, 28.75 mg kg^-1^ available N, and 8.75 mg kg^-1^ available P. The pH of the soil was 7.25. Its texture was loam, with 46% sand, 33.3% silt, and 20.3% clay [29]. After 30 days, the plant was harvested and separated into roots and shoots. The roots were quickly rinsed with distilled water and gently blotted with filter paper to prevent washing away ZnO-NPs. The fresh weight (FW) was promptly measured and kept at -80 °C. Additional roots and shoots were oven-dried to determine their DW.

### Zinc content

Zinc was quantified following sample digestion, as per the procedure outlined by Humphries [30]. Zinc levels were analyzed via atomic absorption spectrophotometry (Buck Model 210 VGP, USA). Results are expressed as µg FW^− 1^.

The translocation factor (TF) measures zinc transport from roots to shoots [31]. An elevated TF indicates efficient upward Zn transport, which directly drives zinc bioaccumulation in edible arugula shoots. The following equation was used to calculate the TF:

$$\mathrm{TF}_{\mathrm{root-shoot}}=\mathrm{Zn}_{\mathrm{shoot}}/\mathrm{Zn}_{\mathrm{root}}$$

Where ‘Zn shoot’ and ‘Zn root’ denote the shoot and root Zn concentrations, respectively.

### Assessment of the pigments

#### Total photosynthetic pigments

The concentrations of total photosynthetic pigments were determined using a spectroscopic method described by Lichtenthaler [32]. To extract photosynthetic pigments, a fresh leaf sample was treated with 95% ethyl alcohol at 60 °C until it became colorless. The absorption was determined by a spectrophotometer at three wavelengths: 452, 644, and 663 nm. The total pigment content was calculated using the equations after summing chlorophylls *a*, *b*, and carotenoids; results are expressed as mg g^− 1^ FW.

$$\begin{array}{l}{\mathrm{Chlorophyll}\;a=\left(13.36 * \mathrm{A}_\mathrm{663}\right)-\left(5.19*\mathrm{A}_{644}\right)}\\{\mathrm{Chlorophyll}\;b=\left(27.49*\mathrm{A}_{644}\right)-\left(8.12*\mathrm{A}_{663}\right)}\\{\mathrm{Carotenoids}=\left[\left(1000*\mathrm{A}_{452}\right)-\left(2.13*\mathrm{Chl}.a\right)-\left(97.6*\mathrm{Chl}.b\right)\right]/209}\end{array}$$

#### Anthocyanin pigments

The concentrations of anthocyanin pigments were determined using a spectroscopic method described by Mancinelli [33]. Using a solution of methanol with 1% (v/v) HCl, a leaf sample underwent extraction for 48 h at 4 °C. The extracts were filtered, and the anthocyanin concentration was determined by measuring the absorbance at 530 and 657 nm wavelengths. The anthocyanin concentration was measured in mg g^− 1^ FW.

### Hydrogen peroxide scavenging

Hydrogen peroxide scavenging activity was measured using a modified method based on Long et al. [34] and Ravindran and Naveenan [35]. To determine scavenging activity, the sample extract was incubated with H₂O₂ for 30 min at room temperature. The FOX reagent, consisting of methanol, H₂SO₄, butylated hydroxytoluene, ferrous ammonium sulfate hexahydrate, and xylenol orange, was then added. Following vortexing and 30 min of incubation, the absorbance was recorded at 560 nm. The percentage of H₂O₂ scavenging was determined by comparing the absorbance of the sample (As) against the control (Ac):

$$\%\;\mathrm{Scavenging} = \text{Ac - As/Ac}\;\mathrm{X}\;100$$

### Assessment of cell membrane stability

The stability of cell membranes was evaluated using three techniques: electrical conductivity (EC), potassium (K) leakage, and ultraviolet (UV)-absorbing metabolite leakage.

### Electrical conductivity

The EC method was used to determine membrane damage by measuring the leakage of electrolytes [36]. During a 24-hour period, leaf discs were immersed in 10 mL of deionized water at 10 °C. The initial electrical conductivity (EC1) was measured at 25 °C. After autoclaving for 15 min, the electrical conductivity (EC2) was determined. The percentage of membrane damage was calculated as electrical conductivity.

$$\text{Electrical conductivity}\left(\%\right)=\left(\mathrm{EC1/EC2}\right)\times100$$

### Potassium leakage

Potassium ion leakage was measured using a flame photometer before and after sterilization [37]. The K leakage was measured in the same EC solution.

### UV-absorbing metabolite leakage

The UV-absorbing metabolites solution was measured using the method described by Navari-Izzo et al. [38]. The UV-absorbing metabolites were measured in the same EC solution.

### Measurement of malondialdehyde concentration

The malondialdehyde (MDA) content, a marker of lipid peroxidation, was quantified using the thiobarbituric acid (TBA) method [39]. The sample was homogenized in 0.1% trichloroacetic acid (TCA) to extract cellular components and centrifuged at 10,000 rpm for 10 min at 4 °C. The supernatant was mixed with a solution containing 20% TCA and 0.5% TBA and heated at 95 °C for 30 min. The reaction mixture was cooled down and centrifuged at 10,000 rpm for 15 min, and the absorbance of the supernatant was measured at 532 nm using a spectrophotometer. We calculated the MDA concentration in mM g⁻¹ FW, applying an extinction coefficient of 155 mM⁻¹ cm⁻¹.

### Determination of ascorbic acid concentration

Ascorbic acid (AsA) concentration was measured using the method described by Jagota and Dani [40]. The frozen sample was crushed in liquid nitrogen, homogenized with 5% trichloroacetic acid (TCA), and centrifuged at 10,000 rpm for 15 min at 4 °C. After adding 10% TCA to the supernatant, it was incubated for five min in an ice bath. The mixture was diluted with distilled water, and then Folin reagent (0.2 M) was added and left for 10 min of incubation to allow for complete color development. The absorbance was recorded at 760 nm, and the results were expressed in mg g⁻¹ FW.

### Analysis of total phenolic compounds

The Folin-Ciocalteu method [41] was used to quantify phenolic compounds. The sample underwent extraction in 50% methanol at 80 °C for 90 min and was then centrifuged at 14,000 rpm for 15 min. The methanol extract was diluted with distilled water, treated with a Na_2_CO_3_ solution, and subsequently mixed with Folin-Ciocalteu’s phenol reagent (2 N). After 20 min of room temperature incubation, spectrophotometry was used to measure the mixture’s absorbance at 725 nm. The total phenolic results, calculated as mg g⁻¹ FW, were quantified using a gallic acid calibration curve.

### Proline determination

The acid-ninhydrin method [42] was used to quantify proline concentration. The sample was homogenized with 3% sulfosalicylic acid, and the resulting homogenate was centrifuged for 10 min at 10,000 rpm. The supernatant was then mixed with glacial acetic acid and the acid-ninhydrin reagent. Toluene was used to extract the color, and a spectrophotometer was used to measure it at 520 nm. The results, expressed in mg g^− 1^ FW, were quantified using a proline calibration curve.

### Cysteine determination

The cysteine (Cyst) concentration was measured using the ninhydrin-acid method [43]. The leaf sample was homogenized in 5% perchloric acid and then centrifuged at 13,000 rpm for 20 min at 4 °C. The supernatant was mixed with acid-ninhydrin reagent, heated for ten min, and then rapidly cooled to stop the reaction. The absorbance of the resulting solution was measured at 560 nm using a spectrophotometer. The results, expressed in mg g^− 1^ FW, were quantified using a Cyst calibration curve.

### Glutathione quantification

The glutathione (GSH) concentration was measured using the Anderson et al. [44] technique. The leaf sample was pulverized in liquid nitrogen, homogenized with sulfosalicylic acid, and then centrifuged at 12,000 rpm for 20 min at 4 °C. The supernatant was mixed with a solution of 0.1 M PPB (pH 7.0), EDTA, and 5,5’-dithiobis-2-nitrobenzoic acid (DTNB) after an incubation time of five min. The absorbance of the resulting solution was measured at 412 nm using a spectrophotometer. The results, expressed in mg g^− 1^ FW, were quantified using a GSH calibration curve.

### Extraction of antioxidant enzymes

Leaf tissues were ground in liquid nitrogen and homogenized in 100 mM phosphate buffer (PPB; pH 7.8) containing 0.1 mM ethylenediaminetetraacetic acid (EDTA) and polyvinylpyrrolidone. The mixture was centrifuged at 18,000 rpm for 10 min at 4 °C, then the supernatant was collected and used for evaluating the activities of all tested enzymes, amino acids, and soluble protein analyses.

### Free amino acid quantification

The method developed by Moore and Stein [45] was used to measure free amino acids. The enzyme extract was mixed with SnCl₂ reagent, heated in a water bath for 20 min, and then diluted with diluent solvent. The absorbance of the resulting solution was measured at 570 nm using a spectrophotometer. The results, expressed in mg g^− 1^ FW, were quantified using a glycine calibration curve.

### Soluble protein quantification

Soluble protein concentration was measured using the Lowry et al. [46] technique. The enzyme extract was mixed with an alkaline solution (Na₂CO₃ and CuSO₄·5 H₂O), then Folin-Ciocalteu’s reagent was added; it was left to sit at room temperature for half an hour. The absorbance of the resulting solution was measured at 750 nm using a spectrophotometer. The results, expressed in mg g^− 1^ FW, were quantified using a bovine serum albumin calibration curve.

### Superoxide dismutase (SOD; EC 1.15.1.1)

Superoxide dismutase (SOD) activity was measured using the epinephrine method [47]. The enzyme extract was mixed with a solution containing 50 mM sodium carbonate buffer (pH 10.2) and 0.1 mM EDTA. The absorbance at 480 nm was monitored for one minute. The enzyme activity was then calculated by dividing the change in absorbance by the protein concentration (mg protein⁻¹) and the time interval (minute).

### Catalase (CAT; EC 1.11.1.6)

Catalase activity was assayed as described by Aebi [48]. The reaction medium contained 50 mM PPB (pH 7), 10 mM H_2_O_2_, and enzyme extract. The absorbance at 240 nm was monitored for one minute. The enzyme activity was calculated by dividing the change in absorbance by the protein concentration (mg protein^− 1^) and the time interval (minute).

### Peroxidase (POD; EC 1.11.1.7)

Peroxidase activity was evaluated as described by Tatiana et al. [49]. The reaction medium consisted of 30 mM PPB (pH 7), 6.5 mM H_2_O_2_, 1.5 mM guaiacol, and enzyme extract. The absorbance at 470 nm was monitored for one minute. The enzyme activity was then calculated by dividing the change in absorbance by the protein concentration (mg protein^− 1^) and the time interval (minute).

### Statistical analysis

The results were obtained from four biological samples, averaging three technical replicates. The standard deviation (SD) was included to indicate data variability. Statistical analysis was conducted using SPSS version 22. One-way analysis of variance (ANOVA) followed by Tukey’s HSD test (*P* ≤ 0.05) was used to determine significant differences among treatments. Eta-squared (η^2^) is an effect size measure that quantifies the proportion of variance explained by a treatment. Eta squared was calculated as the effect’s sum of squares (SS_Effect_) divided by the total sum of squares (SS_Total_). Significant differences between ZnO-NP treatments with or without HRW were denoted as follows: *P ≤ 0.05, **P ≤ 0.01, ***P ≤ 0.001. The *p*-values were obtained using an Excel t-test.

## Results

### ZnO-NPs characterization

**Fig. 1:**
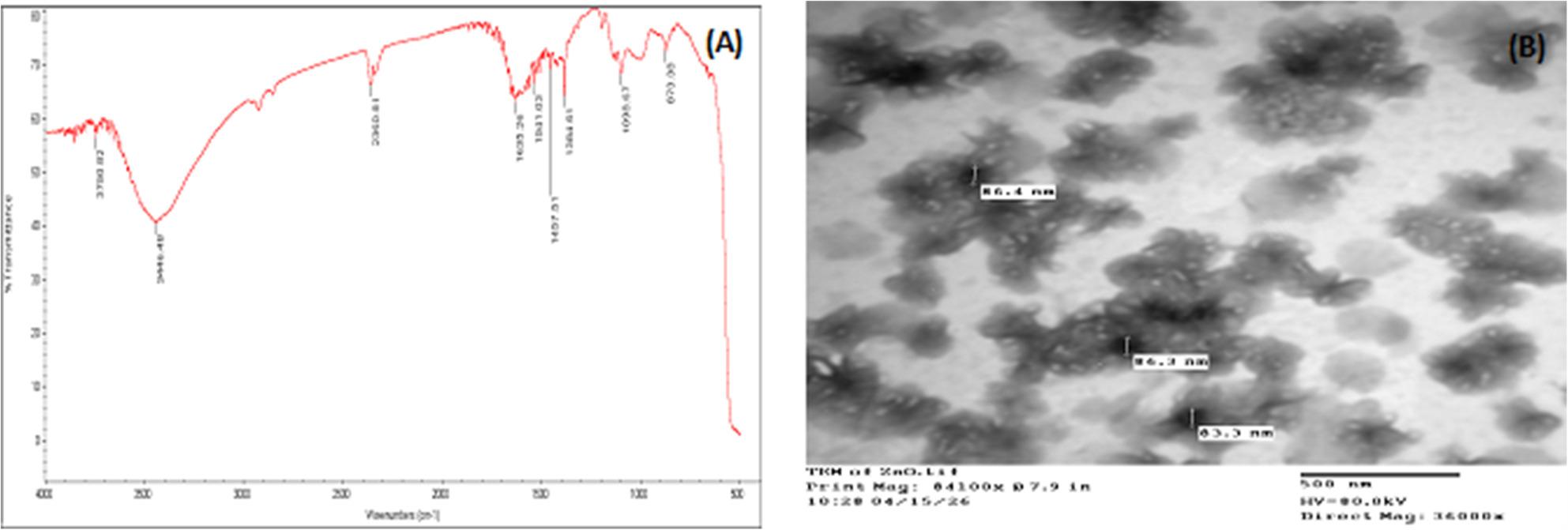
The FTIR spectrum of ZnO-NPs (**A**) and the transmission electron microscopy (TEM) image of the synthesized ZnO-NPs (**B**) are presented. The micrograph was captured at a direct magnification of 36,000x with an accelerating voltage of 80.0 kV

The FTIR spectrum showed several characteristic absorption bands, confirming the formation of ZnO-NPs and the presence of surface functional groups (Fig. 1A). The broad peak centered at 3446.48 cm^− 1^ is attributed to the stretching vibrations of adsorbed water molecules or surface hydroxyl (-OH) groups. Surface-adsorbed moisture was further evidenced by the H-O-H bending vibration at 1635.26 cm⁻¹, a common feature in NPs with high surface-to-volume ratios. The small doublet near 2360 cm^− 1^ is attributed to the presence of ambient carbon dioxide (CO_2_) in the spectrophotometer chamber. The peaks in the 1541 –1384 cm^− 1^ region and the sharp peak at 873.38 cm^− 1^ are typically associated with the asymmetric stretching and out-of-plane bending of carbonate (CO_3_^2−^) groups. These often form due to the absorption of atmospheric CO_2_ during synthesis or sample handling. The peak at 1098.67 cm^− 1^ is attributed to C-O stretching, likely arising from precursor residues left after the washing process. In the fingerprint region, a sharp and prominent peak was detected near 500 cm^− 1^, which is characteristic of the ZnO stretching vibration mode, confirming the successful formation of ZnO.

The morphological characteristics and particle size distribution of the ZnO-NPs were investigated using transmission electron microscopy (TEM). TEM micrographs of the ZnO-NPs reveal quasi-spherical and irregular shapes with evident agglomeration (Fig. 1B). The particles appear as clusters of varying densities due to high surface energy. Manual diameter measurements performed on representative individual particles yielded values of 86.4 nm, 86.3 nm, and 83.3 nm, indicating a relatively uniform size distribution within this specific viewing field. The average particle size is approximately 85.3 nm. The 500 nm scale bar confirms that the structures are well within the nanometer range.

### Tolerance index

**Fig. 2:**
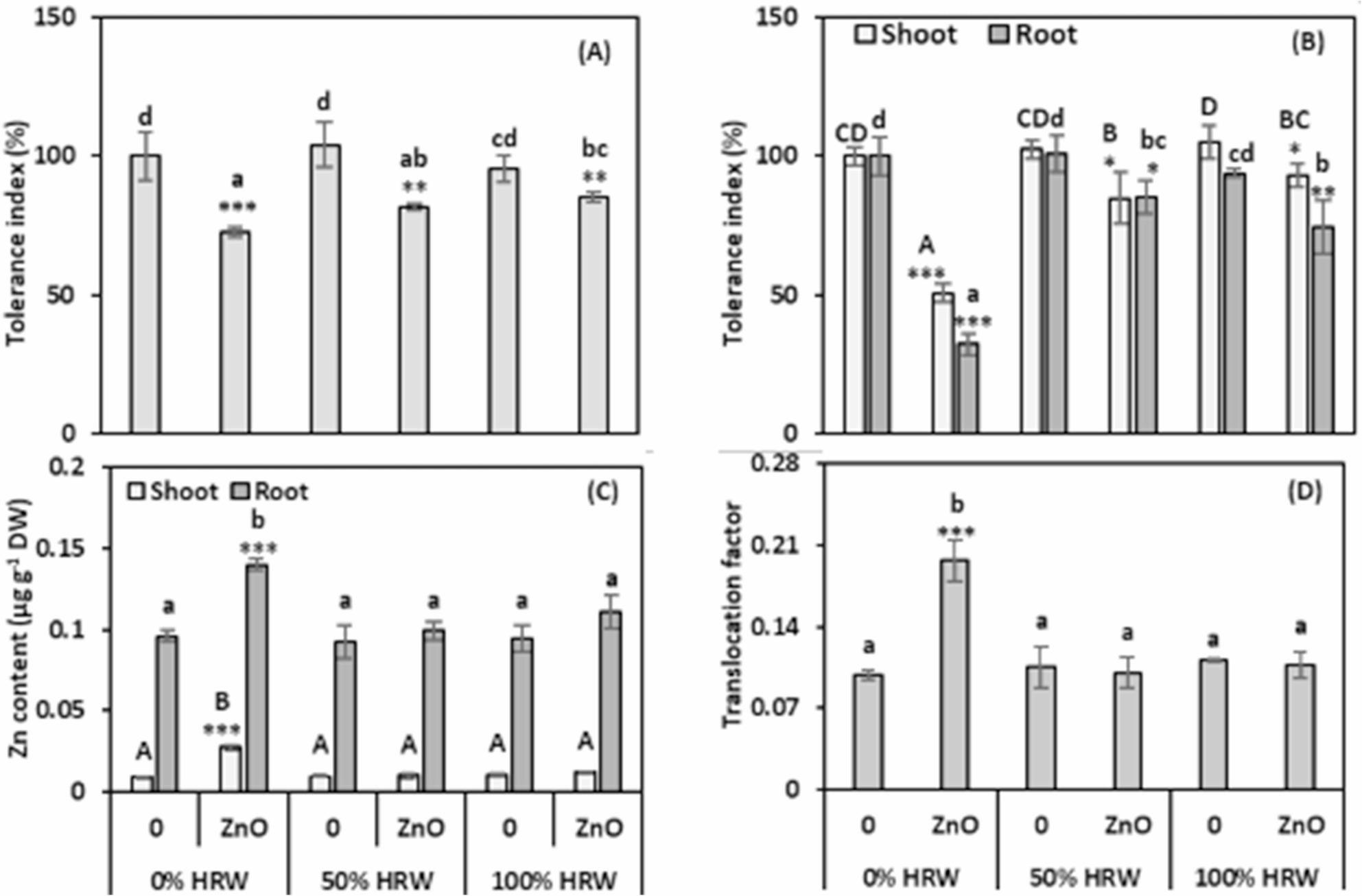
The tolerance index (TI) of seedlings (**A**), TI of shoots and roots (**B**), zinc concentration (Zn) of shoots and roots (**C**), and translocation factor (**D**) of *Eruca sativa* plants under the influence of 150 mg L^− 1^ ZnO-NPs and hydrogen-rich water (HRW; 50% and 100%). Data are the means ± SD (*n* = 4). The different letters, uppercase for TI of shoots and Zn concentration of shoots and lowercase for TI of seedlings, TI of roots, Zn concentration of roots, and translocation factor, indicate significance (one-way ANOVA and Tukey HSD post hoc test). Asterisks indicate significant differences between treatments with or without ZnO-NPs (t-test; *P *≤* *0.05;* **P *≤* 0.01; ***P *≤* 0.001)

This study investigated the effect of ZnO-NPs on arugula seedlings at different growth stages (early and late) by evaluating their TI (Fig. 2A-B). Compared to control seedlings, ZnO-NP exposure resulted in a significant reduction in the seedling TI. This reduction was 27.58% in early-stage seedlings, 49.44% in late-stage shoots, and 67.84% in late-stage roots. HRW treatments effectively counteracted the harmful effects of ZnO-NPs on seedling tolerance. At 50% and 100% HRW concentrations, the early-stage TI increased by 12.37% and 17.74%, respectively, compared to ZnO-NP-stressed plants. HRW significantly increased both shoot and root TIs. The increases were notably more substantial in roots (165.49% and 130.97%) compared to shoots (67.73% and 83.95%). Compared to the control, standalone applications of HRW at 50% and 100% concentrations showed no significant effect on seedling, shoot, or root TI. A notable finding was that the combination of 100% HRW and ZnO-NPs resulted in the greatest effect size (η² = 0.125) on the seedling TI. Statistical analysis revealed that the 100% HRW and ZnO-NP combination produced the greatest effect size (η² = 0.162) on the shoot TI. Differing from the shoot TI results, the 50% HRW and ZnO-NPs combination had the greatest effect size (η^2^ = 0.204) on the root TI.

### Zinc accumulation and translocation

To evaluate how HRW (50% or 100% saturation) alleviates Zn stress and promotes plant development, Zn accumulation was measured in arugula roots and shoots (Fig. 2C). Zinc stress significantly elevated Zn concentrations in the shoots and roots by 192.03% and 46.09%, respectively, relative to the unstressed control plants. Applications of 50% and 100% HRW significantly reduced Zn accumulation in arugula plants. Specifically, Zn levels in the shoots dropped to 36.34% and 43.18%, while root accumulation decreased to 29.11% and 20.78%, respectively, compared to Zn-stressed plants. Notably, Zn accumulation in arugula was significantly lower in the shoot system compared to the root system. HRW treatments did not meaningfully alter shoot or root Zn levels under non-stress conditions. Significant interactions were observed between 50% HRW and ZnO-NP treatments regarding Zn accumulation in roots (ƞ² = 0.206) and shoots (ƞ² = 0.260), respectively.

The TF was evaluated to determine the efficiency of Zn transport from roots to shoots under ZnO-NP stress, both in the presence and absence of HRW treatments (Fig. 2D). Exposing plants to ZnO-NPs in the absence of HRW led to a highly significant elevation in the TF (*P* *≤* 0.001), representing a 100.06% increase over the control group. Notably, the application of HRW effectively mitigated this stress-induced increase. Under both 50% and 100% HRW treatments, the TF in the ZnO-exposed groups decreased to baseline levels. The interaction between 50% HRW and ZnO-NPs significantly influenced the root-to-shoot TF (ƞ² = 0.292).

### Pigments

#### Total photosynthetic pigments

**Fig. 3:**
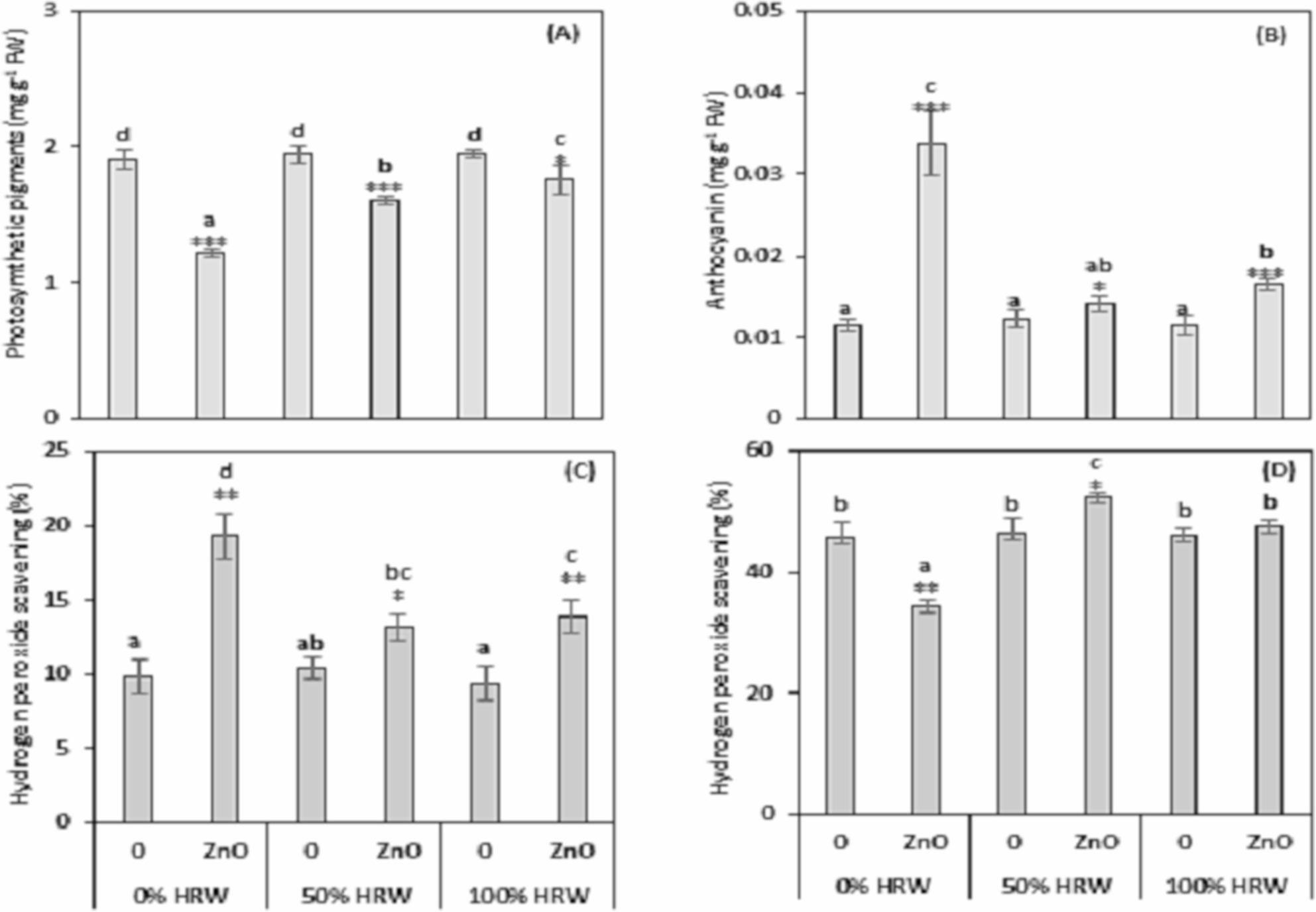
The total photosynthetic pigments (**A**), anthocyanin (**B**), hydrogen peroxide scavenging of seedlings (**C**), and leaves (**D**) of *Eruca sativa* plants under the influence of 150 mg L^− 1^ ZnO-NPs and hydrogen-rich water (HRW; 50% and 100%). Statistical analysis symbols as indicated in Fig. 2

Analyzing photosynthetic pigments provides an efficient method to assess the impact of ZnO-NPs on arugula’s late-stage leaves (Fig. 3A). ZnO-NPs negatively influenced photosynthetic pigment concentrations, causing a 35.86% decrease compared to the control. HRW treatments effectively counteracted the harmful effects of ZnO-NPs on leaf photosynthetic pigment content. At 50% and 100% HRW, photosynthetic pigment concentrations increased by 30.96% and 43.78%, respectively, compared to ZnO-NP stressed leaves. Under non-stress conditions, HRW treatments showed no substantial impact on photosynthetic pigment levels. The interaction between 100% HRW exposure and ZnO-NP application significantly impacted pigment levels (ƞ^2^ = 0.157).

#### Anthocyanin pigments

Leaf anthocyanin concentrations were dramatically elevated by ZnO-NP stress, showing a 194.97% increase relative to controls (Fig. 3B). HRW treatments mitigated the ZnO-NP-induced increase in leaf anthocyanin concentrations. 50% and 100% HRW reduced anthocyanin by 58.01% and 50.74%, respectively, compared to leaves treated with ZnO-NPs alone. HRW did not increase anthocyanin in unstressed plants. The 50% HRW and ZnO-NP treatment combination exhibited the highest effect on leaf anthocyanin concentration, as indicated by an eta-squared value of 0.255.

### Hydrogen peroxide scavenging

Hydrogen peroxide scavenging is a key antioxidant process that neutralizes H_2_O_2_ into water, shielding cells from the deleterious effects of oxidative stress. Hydrogen peroxide scavenging activity in arugula plants subjected to Zn stress and HRW treatments is shown in Fig. 3C-D. Seedlings displayed lower H_2_O_2_ scavenging activity compared to mature arugula leaves (Fig. 3C-D). Seedlings treated with ZnO-NPs exhibited a 96.69% increase, approximately two-fold relative to the control group. The addition of 50% and 100% HRW to ZnO-NP-treated seedlings significantly reduced H_2_O_2_ scavenging activity by 31.79% and 28.29%, respectively, compared to those treated with ZnO-NPs alone.

Leaf H_2_O_2_ scavenging activity dropped by 24.59% under ZnO-NP stress compared to control conditions (Fig. 3D). The decline in leaf H_2_O_2_ scavenging activity caused by ZnO-NPs was effectively counteracted by the application of HRW. HRW supplementation (50% and 100%) improved H_2_O_2_ scavenging activity by 52.32% and 37.63%, respectively, over the ZnO-NP-stressed group. In the absence of stress, HRW treatments exerted no significant effect on H_2_O_2_ scavenging activity in either seedlings or leaves. The 50% HRW and ZnO-NP treatment yielded the highest impact on H_2_O_2_ scavenging, showing stronger effects in leaves (ƞ² = 0.294) than in seedlings (ƞ² = 0.166).

### Membrane integrity

**Fig. 4:**
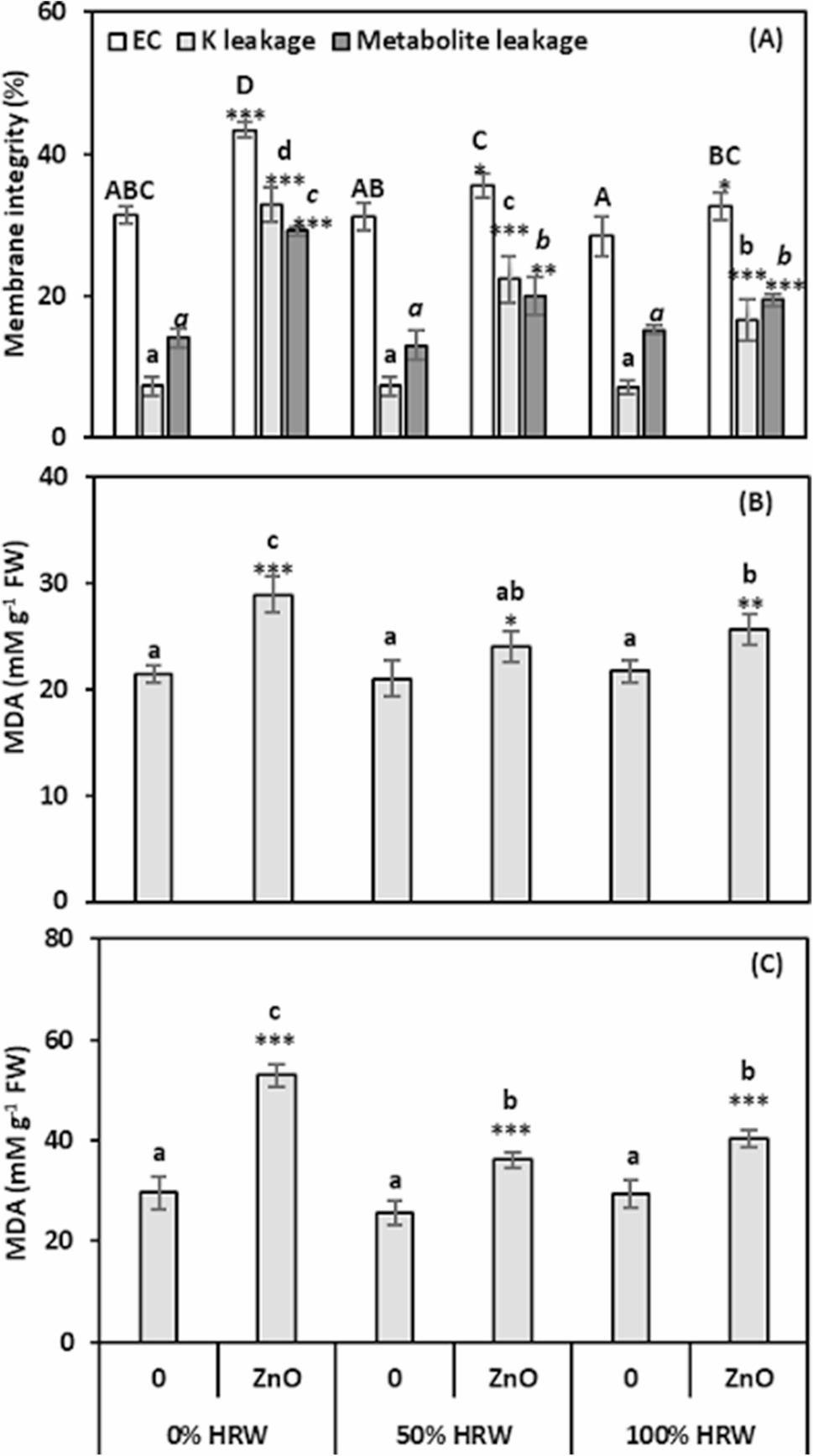
The cell membrane stability index of leaves [electrical conductivity (EC), K leakage, and metabolite leakage] (**A**), malondialdehyde (MDA) of seedlings (**B**), and leaves (**C**) of *Eruca sativa* plants under the influence of 150 mg L^− 1^ ZnO-NPs and hydrogen-rich water (HRW; 50% and 100%). The different letters, uppercase for EC, lowercase for K leakage and MDA, and small italic for metabolite leakage, indicate significance. Statistical analysis symbols as indicated in Fig. 2

Leaf membrane damage was evaluated by quantifying EC alongside K⁺ and UV-absorbing metabolite leakage (Fig. 4A). Relative to controls, ZnO-NP stress significantly elevated leaf (late stage) EC, K leakage, and UV-absorbing metabolite leakage, with increases of 38.38%, 352.19%, and 106.56%, respectively. HRW treatments significantly reduced EC, K ion leakage, and UV-absorbing metabolite leakage compared to plants treated with ZnO-NPs alone. Treatment with 100% HRW resulted in a significant reduction in EC, K leakage, and UV-absorbing metabolite leakage, with decreases of 24.68%, 49.19%, and 33.18%, respectively, compared to ZnO-NPs alone. HRW treatments without stress did not significantly alter EC, K leakage, and UV-absorbing metabolite leakage compared to the control. A two-way ANOVA demonstrated that the combined application of 100% HRW and ZnO-NPs yielded the highest effect sizes for EC, potassium leakage, and UV-absorbing metabolite leakage, with ƞ^2^ values of 0.106, 0.129, and 0.173, respectively.

The study investigated the influence of HRW treatments on ZnO-NP-induced oxidative damage by quantifying MDA content, a marker of lipid peroxidation, in seedlings and leaves (Fig. 4B-C). Relative to controls, ZnO-NP exposure significantly elevated MDA accumulation, with increases of 34.63% in seedlings (early stage) and 78.88% in leaves (late stage). 50% and 100% HRW treatments effectively mitigated the increase in MDA concentrations caused by ZnO-NPs. Treatment with 50% HRW resulted in a 16.90% decrease in seedling MDA and a 31.76% decrease in leaf MDA, while 100% HRW resulted in 11.28% and 23.66% decreases, respectively. Treatment with HRW alone did not significantly affect MDA concentrations in seedlings and leaves compared to the untreated control. The two-way analysis revealed the highest size effect for reducing MDA levels in seedlings (ƞ^2^ = 0.115) and leaves (ƞ^2^ = 0.087) with the combined treatment of 50% HRW and ZnO-NPs.

### Thiol compounds

**Fig. 5:**
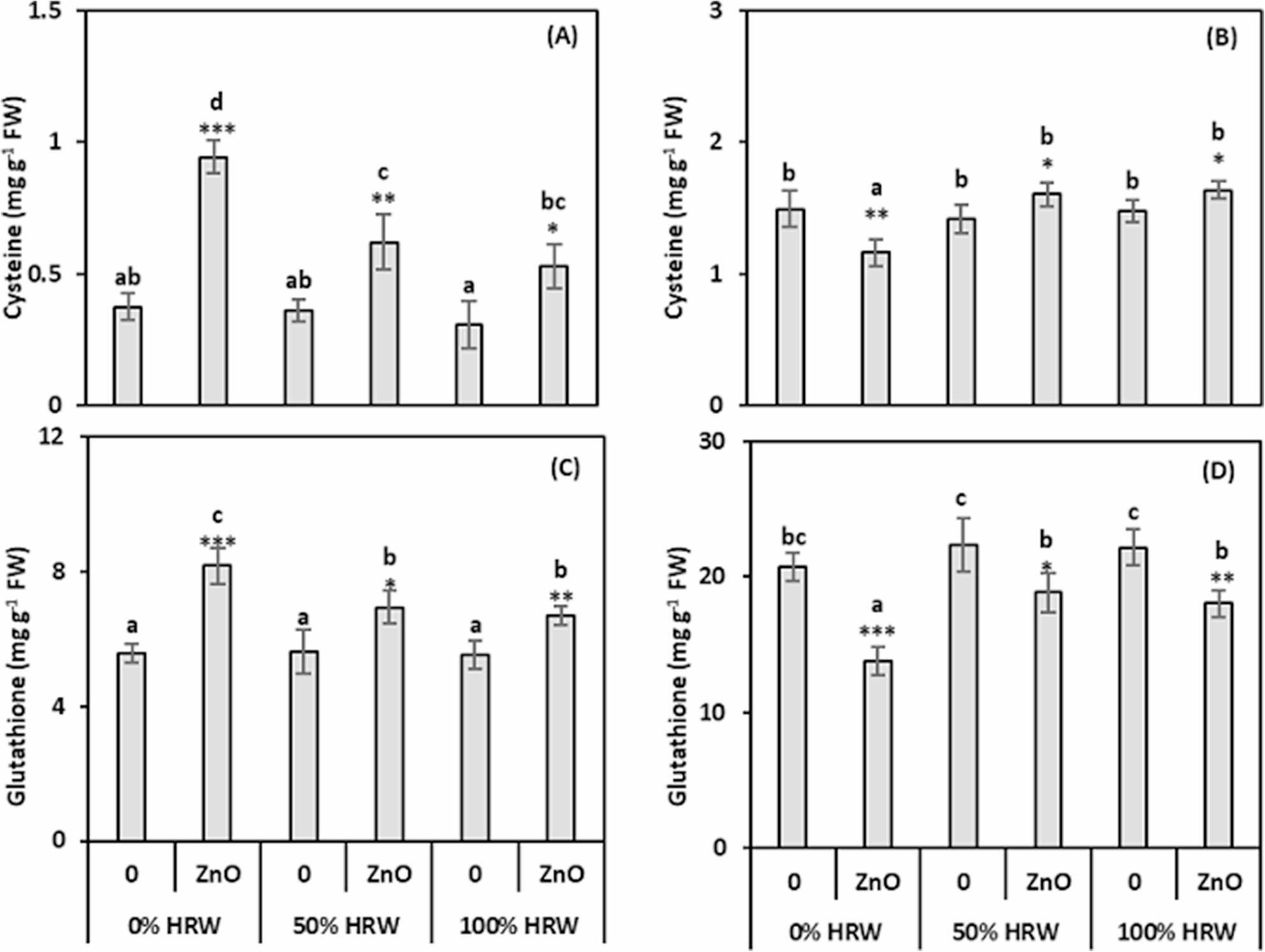
Cysteine of seedlings (**A**), leaves (**B**), glutathione of seedlings (**C**), and leaves (**D**) of *Eruca sativa* plants under the influence of 150 mg L^− 1^ ZnO-NPs and hydrogen-rich water (HRW; 50% and 100%). Statistical analysis symbols as indicated in Fig. 2

To test whether HRW-induced stress mitigation involves enhanced thiol accumulation, Cyst and GSH were quantified in ZnO-NP-stressed leaves and seedlings. ZnO-NPs induced variable changes in Cyst levels across seedlings and leaves (Fig. 5A-B). Notably, Cyst content was higher in leaves than in seedlings. Relative to the control, ZnO-NP treatment led to a 152.68% increase in Cyst concentrations in seedlings. Compared to ZnO-NP -treated seedlings, 50% and 100% HRW treatments resulted in a 34.56% and 44.28% decrease in Cyst accumulation, respectively. On the other hand, ZnO-NPs significantly decreased leaf Cyst concentrations by 22.29% compared to the control (Fig. 5B). The application of HRW significantly enhanced Cyst accumulation in leaves exposed to ZnO-NPs. Compared to ZnO-NP -treated plants alone, Cyst concentrations increased by 38.28% and 41.12% at 50% and 100% HRW, respectively. HRW treatment did not affect Cyst concentrations in control plants, regardless of developmental stage. For seedlings, 100% HRW with ZnO-NPs had the largest effect on Cyst concentrations (ƞ^2^ = 0.112), but for leaves, 50% HRW with ZnO-NPs showed the greatest influence (ƞ^2^ = 0.305).

GSH levels in seedlings and leaves exhibited variable changes in response to ZnO-NPs (Fig. 5C-D). Seedlings had a lower GSH content than leaves. Seedlings treated with ZnO-NPs exhibited a 47.04% greater GSH concentration than the control. HRW treatments (50% and 100%) led to 15.03% and 18.01% lower GSH accumulation in seedlings, respectively, compared to those treated with ZnO-NPs. ZnO-NPs significantly diminished leaf GSH concentrations by 33.33% compared to the control (Fig. 5D). Applying HRW to ZnO-NP-treated leaves significantly enhanced GSH accumulation. Specifically, 50% and 100% HRW increased GSH concentrations by 36.48% and 30.84%, respectively, relative to ZnO-NP treatment alone. GSH concentrations in control seedlings remained unchanged following HRW treatment across all developmental stages. The most pronounced effect on GSH concentrations was observed in seedlings treated with 100% HRW and ZnO-NPs (ƞ^2^ = 0.106) and in leaves treated with 50% HRW and ZnO-NPs (ƞ^2^ = 0.057).

### Ascorbic acid

**Fig. 6:**
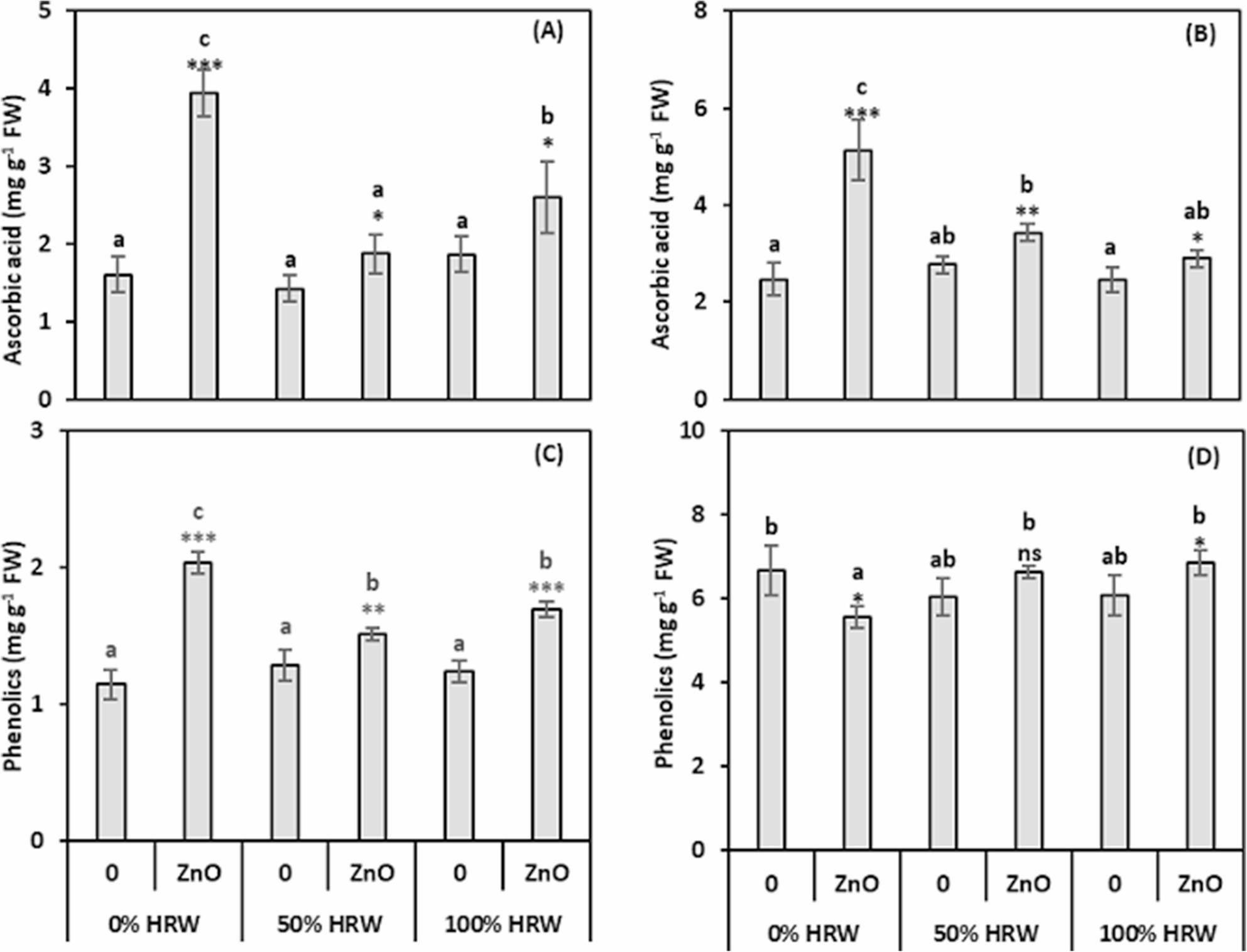
Ascorbic acid of seedlings (**A**), leaves (**B**), total phenolics of seedlings (**C**), and leaves (**D**) of *Eruca sativa* plants under the influence of 150 mg L^− 1^ ZnO-NPs and hydrogen-rich water (HRW; 50% and 100%). Statistical analysis symbols as indicated in Fig. 2

Ascorbic acid content was quantified to determine whether its concentration fluctuations under ZnO-NP stress are linked to HRW-mediated protection. Ascorbic acid concentrations were higher in ZnO-NP-treated seedlings and leaves than in the control (Fig. 6A-B). Ascorbic acid levels were elevated in leaves relative to seedlings. Seedlings treated with ZnO-NPs showed a substantial 146.09% increase in AsA compared to a 108.12% increase in leaves. In ZnO-NP-treated plants, HRW treatments significantly reduced AsA. Specifically, 50% HRW decreased levels by 52.59% in seedlings and 33.17% in leaves, while 100% HRW decreased levels by 34.29% and 43.63%, respectively. Ascorbic acid concentrations were maintained in control plants following HRW treatment. Analysis of effect size (ƞ²) revealed that 50% HRW with ZnO-NPs had the greatest impact on seedling AsA (ƞ² = 0.208), while 100% HRW with ZnO-NPs had the largest effect on leaf AsA (ƞ² = 0.233).

### Total phenolic compounds

Phenolic compound concentrations varied in response to ZnO-NPs and HRW across seedlings and leaves (Fig. 6C-D). Phenolic content was higher in leaves compared to seedlings. ZnO-NP-treated seedlings had a 77.86% higher phenolic concentration than the control. Phenolic accumulation was decreased by 25.62% and 16.78% in seedlings treated with ZnO-NPs plus 50% or 100% HRW, respectively, compared to seedlings treated with ZnO-NPs alone. ZnO-NPs significantly lowered leaf phenolic concentrations by 16.62% compared to the control (Fig. 6D). The HRW application effectively mitigated the negative effect of ZnO-NPs on leaf phenolic accumulation. Compared to ZnO-NP treatment alone, phenolic concentrations increased by 19.13% (50% HRW) and 23.18% (100% HRW). Phenolic compound levels remained unchanged in seedlings and leaves following HRW treatments in the absence of stress. The maximum effect on phenolic levels was observed in seedlings treated with 50% HRW and ZnO-NPs (ƞ² = 0.223) and in leaves treated with 100% HRW and ZnO-NPs (ƞ² = 0.467).

### Proline

**Fig. 7:**
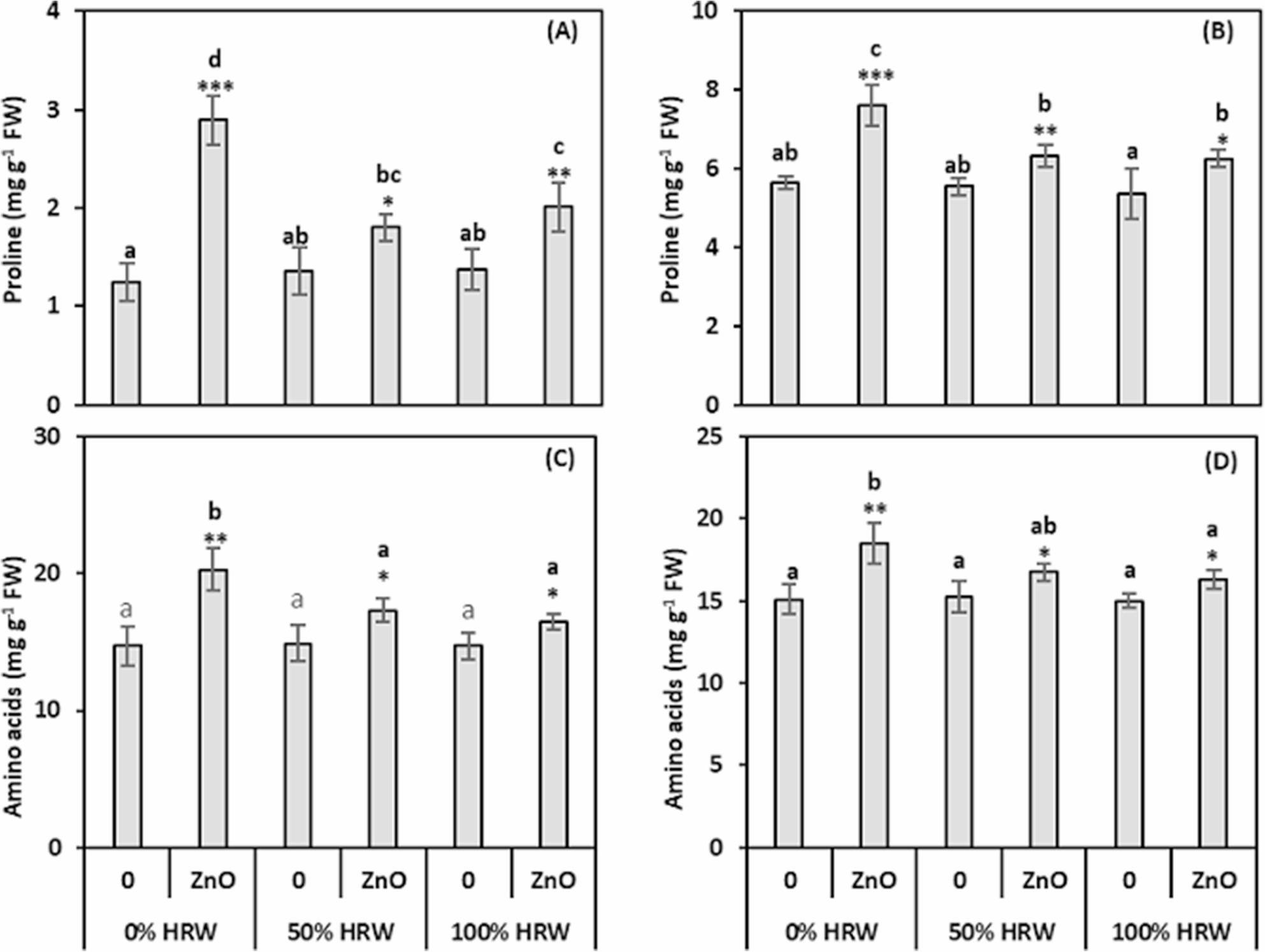
Proline of seedlings (**A**), leaves (**B**), amino acids of seedlings (**C**), and leaves (**D**) of *Eruca sativa* plants under the influence of 150 mg L^− 1^ ZnO-NPs and hydrogen-rich water (HRW; 50% and 100%). Statistical analysis symbols as indicated in Fig. 2

Proline, an osmoprotectant, was quantified in ZnO-NP-stressed arugula seedlings and leaves to assess damage and the protective influence of HRW treatments (Fig. 7A-B). Seedlings had a lower proline content than leaves. Seedlings treated with ZnO-NPs showed a 132.12% increase in proline, while leaves showed a 34.84% increase compared to the control. HRW treatments (50% and 100%) decreased proline concentrations in ZnO-NP-treated plants. The reductions were 37.64% and 30.40% for seedlings and 16.72% and 17.79% for leaves, respectively. HRW by itself didn’t cause proline levels to rise in seedlings or leaves beyond what was seen in the control plants. Treatment with 50% HRW and ZnO-NPs had the highest size effect on proline concentration in both seedlings and leaves (ƞ² = 0.202 and 0.104, respectively).

### Other free amino acids

Amino acid accumulation in arugula tissues was evaluated under ZnO-NP and HRW treatments to assess its contribution to HM tolerance. Amino acid concentrations were significantly elevated by ZnO-NP stress, increasing by 37.65% in seedlings and 22.70% in leaves (Fig. 7C-D). The HRW application significantly attenuated the ZnO-NP-mediated increase in amino acid concentrations within seedling and leaf tissues. Treatment with 50% HRW resulted in a 14.68% reduction in seedling amino acid concentrations and a 9.67% reduction in leaf amino acid concentrations. 100% HRW treatment yielded reductions of 19.02% and 12.16%, respectively. Statistical analysis revealed that the combined treatment of 100% HRW and ZnO-NPs exhibited the highest effect size (ƞ²) on amino acid concentrations, with values of 0.163 for seedlings and 0.136 for leaves.

### Soluble proteins

**Fig. 8:**
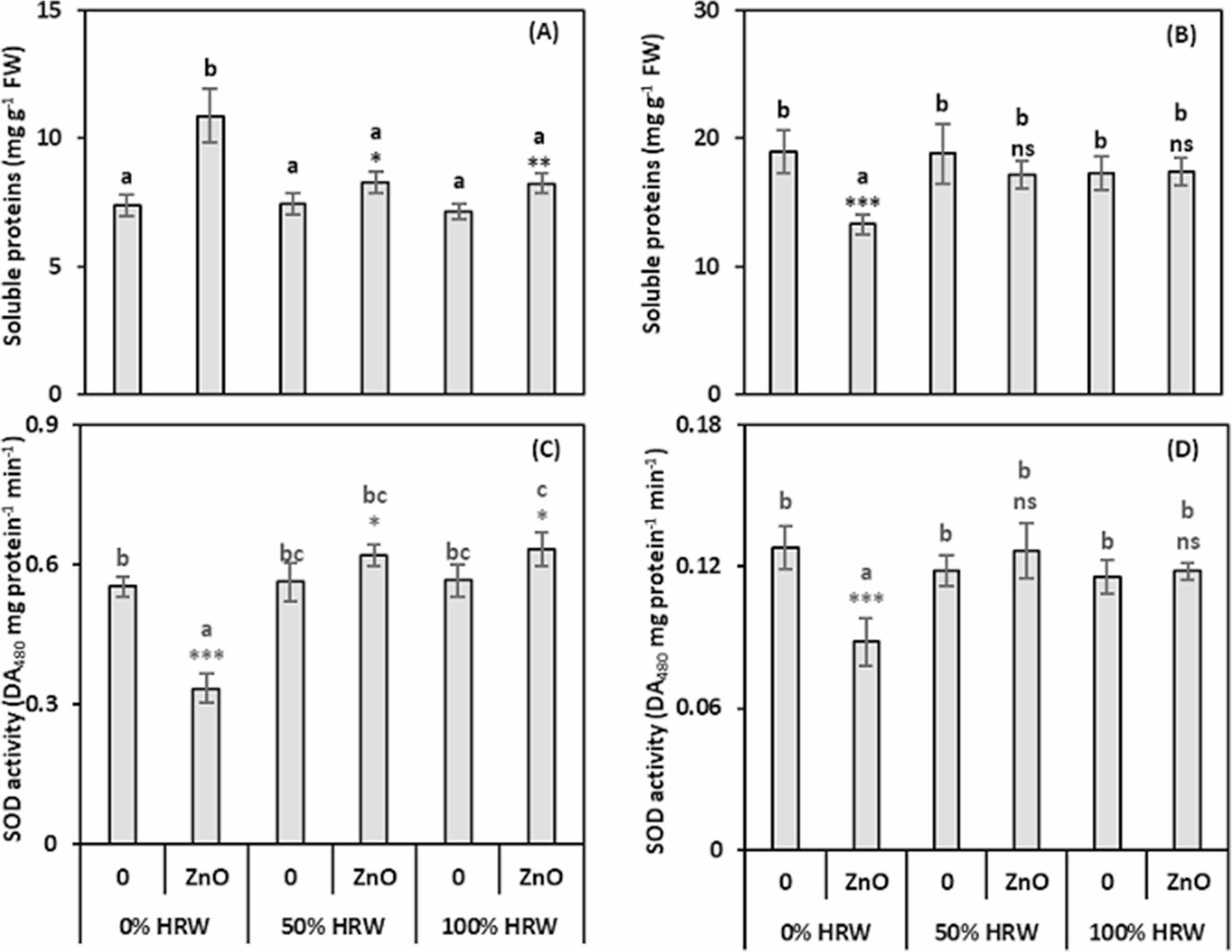
Soluble proteins of seedlings (**A**), leaves (**B**), superoxide dismutase (SOD) of seedlings (**C**), and leaves (**D**) of *Eruca sativa* plants under the influence of 150 mg L^− 1^ ZnO-NPs and hydrogen-rich water (HRW; 50% and 100%). Statistical analysis symbols as indicated in Fig. 2

Soluble protein analysis is necessary to understand the effects of ZnO-NP stress, with/without HRW treatments, on seedlings and leaves. Significant variations in soluble protein concentrations were observed in seedlings and leaves in response to ZnO-NPs and HRW treatments (Fig. 8A-B). Soluble protein content was higher in leaves compared to seedlings. Compared to the control, ZnO-NP treatment raised the concentrations of soluble proteins in seedlings by 47.56%. HRW treatments decreased soluble protein concentrations in ZnO-NP-treated seedlings. Compared to seedlings treated with ZnO-NPs alone, the reduction was 23.76% and 24.34% at 50% and 100% HRW concentrations, respectively. Analysis revealed that ZnO-NPs significantly lowered leaf soluble protein concentrations by 30.07% relative to the untreated control (Fig. 8B). The application of HRW to plants previously treated with ZnO-NPs resulted in an elevation of soluble protein concentrations within the leaves. Compared to plants treated with ZnO-NPs alone, increases were 29.50% and 31.18% at 50% and 100% HRW concentrations, respectively. HRW treatments in control seedlings and leaves did not significantly alter soluble protein concentrations compared to absolute controls. Statistical analysis revealed that the greatest effect on soluble protein levels occurred in seedlings treated with 50% HRW and ZnO-NPs (ƞ² = 0.176) and in leaves treated with 100% HRW and ZnO-NPs (ƞ² = 0.381).

### Superoxide dismutase

To assess the impact of ZnO-NPs and HRW on ROS detoxification, we quantified SOD activity in arugula plants under various treatment combinations (Fig. 8C-D). Analysis revealed a lower SOD activity in leaves compared with seedlings. ZnO-NP stress significantly lowered SOD activity, with seedlings showing a more pronounced decrease (39.64%) compared to leaves (31.18%). HRW treatments at 50% and 100% mitigated the negative effects of ZnO-NP stress by increasing SOD activity in seedlings and leaves. Specifically, seedlings exhibited increases of 85.88% and 89.85%, while leaf tissue showed enhancements of 43.94% and 34.01% under the respective HRW concentrations. In the absence of ZnO-NP stress (control seedlings and leaves), HRW treatments did not significantly alter SOD activity relative to the untreated control group. A substantial effect on SOD activity was observed in leaves treated with 50% HRW and ZnO-NPs (η² = 0.437) and in seedlings treated with 100% HRW and ZnO-NPs (η² = 0.239).

### Catalase

**Fig. 9:**
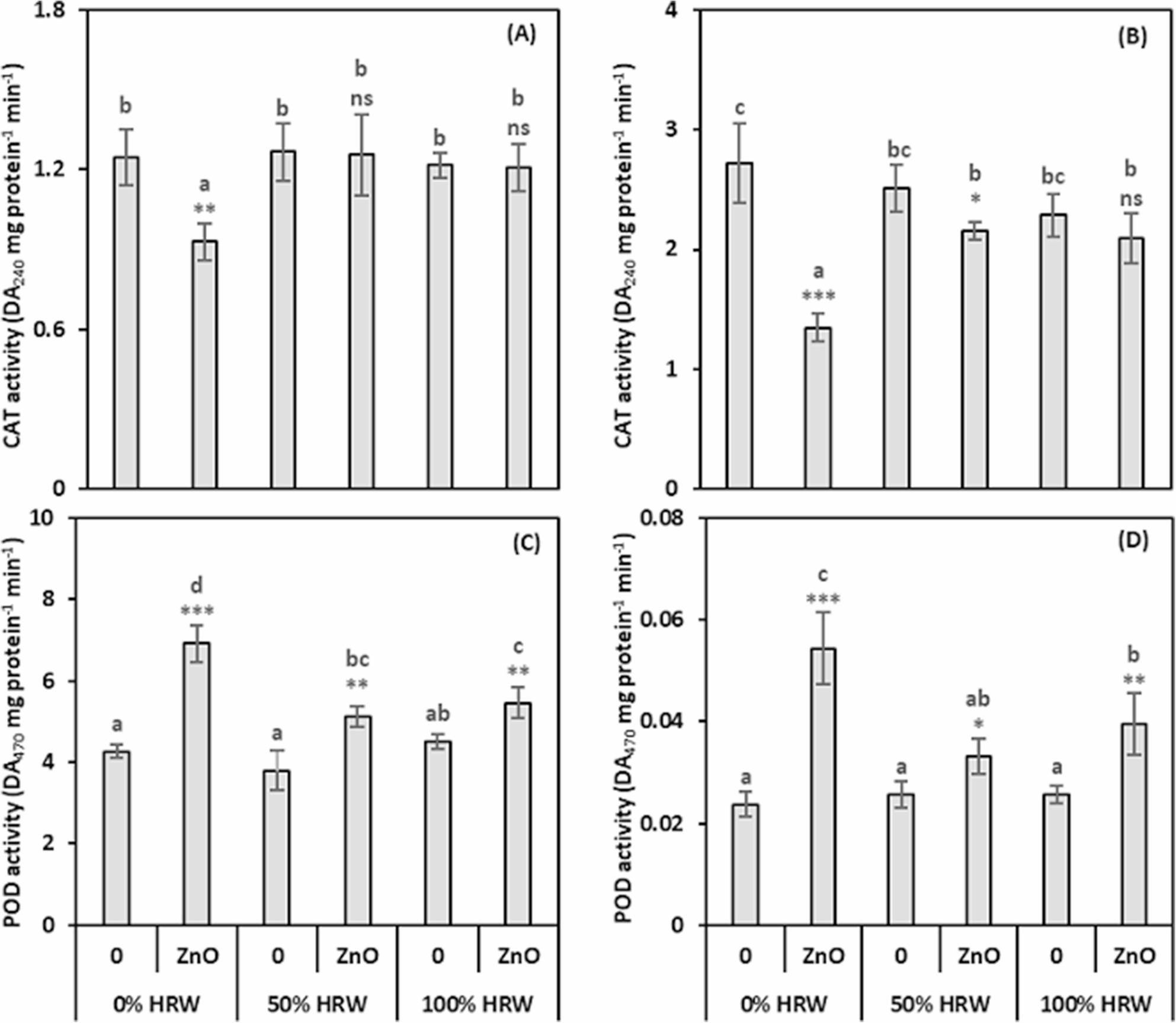
The activity of catalase of seedlings (**A**), leaves (**B**), peroxidase of seedlings (**C**), and leaves (**D**) of *Eruca sativa* plants under the influence of 150 mg L^− 1^ ZnO-NPs and hydrogen-rich water (HRW; 50% and 100%). Statistical analysis symbols as indicated in Fig. 2

The impact of ZnO-NPs and HRW on ROS detoxification was assessed by quantifying CAT activity in arugula plants under various treatment combinations (Fig. 9A-B). CAT activity was elevated in leaves relative to seedlings. ZnO-NP stress caused a significant drop in CAT activity in both leaves and seedlings, but the decrease was much larger in leaves (50.52%) than in seedlings (25.44%). Applying HRW at 50% and 100% concentrations to ZnO-NP stressed plants increased CAT activity in both seedlings and leaves. Seedlings experienced CAT activity increasing by 35.05% (50% HRW) and 29.78% (100% HRW), whereas the corresponding increases in leaf tissue were more pronounced, at 60.02% and 55.10%. HRW treatments, applied to control seedlings and leaves (without ZnO-NP stress), did not induce any significant changes in CAT activity compared to the untreated control group. CAT activity was substantially affected in both seedlings treated with 50% HRW and ZnO-NPs (η² = 0.236) and leaves treated with 100% HRW and ZnO-NPs (η² = 0.328), with the latter showing a larger effect size.

### Peroxidase

To understand how ZnO-NPs and HRW affect ROS detoxification, POD activity was measured in arugula plants under a range of treatment conditions (Fig. 9C-D). Notably, POD activity was lower in leaves than in seedlings. Leaves exhibited a substantially more significant increase in POD activity (129.36%) under ZnO-NP stress compared to seedlings (62.15%), both of which showed significant upregulation relative to control plants. HRW application (50% and 100%) to ZnO-NP stressed plants resulted in decreased POD activity in both tissues. Seedlings showed reductions of 25.78% and 21.01%, respectively, while leaves exhibited more pronounced decreases of 38.82% and 27.27% at the same HRW concentrations. Applied to control seedlings and leaves (without ZnO-NP stress), HRW treatments did not induce significant changes in POD activity compared to the untreated control group. Statistically, the greatest effects on POD activity were in leaves (50% HRW + ZnO-NPs, ƞ² = 0.208) and seedlings (100% HRW + ZnO-NPs, ƞ² = 0.127).

## Discussion

Within nanotechnology, ZnO-NPs represent a rapidly expanding field with diverse scientific applications [50]. However, their inevitable environmental release raises critical concerns regarding ecological toxicity and risks to living systems [51]. While HRW has demonstrated a capacity to alleviate HM toxicity, such as Hg, Al, and Cd [52–54], its efficacy in mitigating engineered nanotoxicity remains largely unexplored. This study addresses this knowledge gap by assessing how HRW modulates defense mechanisms in arugula (*Eruca sativa*) under ZnO-NP stress.

### ZnO-NPs characterization

The FTIR spectrum confirmed the high purity and crystalline nature of the synthesized ZnO-NPs. The strong absorption feature below 600 cm⁻¹ corresponds to the characteristic vibrational modes of diatomic Zn-O in a tetrahedral geometry [55]. The broad O-H stretching band at 3446.48 cm^− 1^ reflects surface hydroxylation or physisorbed water, characteristic of nanomaterials with high surface-to-volume ratios [56]. Slight shifts in peaks associated with functional groups (O-H or C-O) indicate that plant biomolecules successfully acted as both reducing and capping agents during green synthesis [56]. TEM analysis validated these structural traits, revealing a phase-pure, quasi-spherical morphology with an average particle size of approximately 85 nm [55, 56].

### Tolerance index

The TI directly reflects a plant’s susceptibility to environmental pressures [28]. Consistent with established literature, exposure to ZnO-NPs alone significantly decreased arugula biomass and TI [18]. This growth inhibition is tied to altered cellular ultrastructure, structural chromosomal defects, and the arrest of cell division [56–58].

Exogenous HRW treatments (both 50% and 100% saturation) significantly rescued the TI in both roots and shoots. This recovery is partially explained by the “acid growth” hypothesis, wherein plants actively efflux protons (H⁺) to promote cell wall relaxation and elongation [59]. HRW appears to enhance these plasma membrane vesicle activities, stimulating structural expansion under stress [60, 61]. Thus, we deduced that HRW might represent a novel class of gaseous signaling molecules with diverse effects on plant responses to environmental stresses.

### Zinc accumulation and translocation

Exposure to ZnO NPs induced severe Zn hyperaccumulation in both roots and shoots, a profile characteristic of metal toxicity that impairs the photosynthetic apparatus and triggers oxidative cascades [13]. Crucially, the application of HRW at both 50% and 100% saturation levels significantly alleviated this Zn accumulation. This protective effect aligns with a growing body of evidence demonstrating that molecular hydrogen mitigates HM stress (e.g., Hg, Al, and Cd) by restoring redox homeostasis and reducing net metal influx [52–54].

The TF serves as a critical indicator of a plant’s physiological efficiency in mobilizing ions from roots to aerial tissues [31]. In this study, ZnO-NP exposure without HRW doubled the TF compared to the control group (*P* *≤* 0.001), signaling a massive increase in root-to-shoot Zn mobility. Both 50% and 100% HRW applications successfully suppressed this stress-induced surge, returning TF values to the control baseline. The prominent interaction effect between 50% HRW and ZnO-NPs mathematically underscores that HRW acts as an active, regulatory signal rather than a passive chemical buffer. Notably, HRW did not alter Zn distribution or induce deficiencies under non-stressed, normal growth conditions, demonstrating its safety as an agricultural amendment. Because Zn and Cd share overlapping transport pathways, HRW likely downregulates or competitively inhibits key metal transporter proteins, specifically the Heavy Metal ATPase (HMA) and ZRT/IRT-like protein (ZIP) families, that drive xylem loading [31, 51]. Furthermore, excessive ROS generation under ZnO-NP toxicity typically degrades the root endodermis and Casparian strip, breaking down selective apoplastic barriers and allowing unrestricted metal entry into the transpirational stream [13]. HRW fortifies cell wall components and triggers endogenous chelation pathways within root cells, safely trapping free Zn²⁺ or NPs in the root matrix and vacuoles to prevent xylem loading [8, 62]. By rapidly upregulating key enzymatic antioxidants like SOD and CAT, HRW neutralizes the local ROS burst. This preserves root membrane integrity and restores tight, selective control over transpirational xylem transport [34].

### Pigments

Exposure to excess ZnO-NPs induces oxidative stress, damages chloroplast thylakoids and membranes, and inhibits protein synthesis, ultimately suppressing photosynthetic pigment concentration and plant biomass [63]. In this study, ZnO-NPs negatively affected pigment concentrations. However, treatment with HRW significantly increased pigment synthesis, suggesting that HRW effectively mitigates oxidative stress induced by ZnO-NPs. Supporting studies show that HRW enhances photosynthetic activity and chlorophyll content under Al stress [53] and serves as an eco-friendly approach to improve photosynthetic efficiency, antioxidant systems, and overall plant performance [64].

The study observed a positive correlation between elevated ZnO-NP concentrations and increased anthocyanin synthesis. Anthocyanins function as potent antioxidants that scavenge ROS, reduce oxidative damage, and contribute to HM tolerance by chelating metal ions and sequestering them in vacuoles [65–67]. This study discovered a positive relationship between elevated ZnO-NP concentrations and plant anthocyanin synthesis. This finding raises the possibility that anthocyanins are involved in the plant’s reaction to ZnO-NP stress, although more investigation is required to support this theory. HRW appears to interact positively with these stress-response pathways. These findings suggest that HRW may interact with plant ways to respond to stress from ZnO-NPs. To our knowledge, no study suggests that HRW inhibits the synthesis of anthocyanins in plants under ZnO-NP stress. According to Zhang et al. [67], transcriptomic analysis shows that HRW enhances anthocyanin accumulation in UV-A-stressed radish sprouts by modulating the expression of key biosynthetic and regulatory genes.

### Hydrogen peroxide scavenging

Our results demonstrate that the activity of scavenging H_2_O_2_ under ZnO-NP stress is highly tissue- and developmental-stage-specific in arugula. In seedlings, excess Zn triggered a sharp upregulation in scavenging capacity to compensate for elevated ROS production [18]. Conversely, ZnO-NPs caused a significant decline in H_2_O_2_ scavenging activity, indicating that prolonged stress may overwhelm antioxidant systems in fully developed tissues. HRW (both concentrations) significantly reduced this elevated activity, bringing it closer to control levels. In Zn-stressed seedlings, HRW significantly lowered scavenging activity toward control levels, indicating that molecular hydrogen alleviated the primary oxidative burden and reduced the demand for hyper-activated defense systems. In mature leaves, however, HRW rescued the Zn-induced decline, successfully restoring and enhancing H_2_O_2_ scavenging capacity to shield the photosynthetic machinery. Importantly, HRW had no impact under non-stress conditions, highlighting its function as a stress-specific modulator rather than an indiscriminate antioxidant stimulant. These opposing effects demonstrate that HRW helps re-establish redox homeostasis by normalizing antioxidant demands. It reduces excessive ROS production (possibly by limiting Zn uptake/translocation), prevents overactivation of stress responses, and protects photosynthetic tissues. The findings are consistent with previous research showing molecular hydrogen (via HRW) effectively mitigates oxidative stress from HMs (Hg, Al, and Cd) while remaining neutral under normal conditions [52–54].

### Membrane integrity

Membrane stability is a key indicator of plant tolerance to HMs, as these stressors often damage cell membranes, impairing their structural and functional integrity [68]. In this study, ZnO-NP treatment significantly increased membrane permeability in arugula plants. This was evidenced by elevated EC and increased leakage of K⁺ and UV-absorbing metabolites, clear signs of oxidative stress-induced membrane damage. These results are consistent with previous findings, such as increased MDA content in pomegranate calli exposed to ZnO-NPs [56]. Conversely, HRW treatment significantly mitigated this damage, inhibiting the rise in membrane permeability. This protective effect is consistent with findings by Cui et al. [6], where HRW enhanced antioxidant enzyme activity and reduced membrane lipid peroxidation under Cd stress. Two distinct mechanisms likely drive this response: HRW may directly alleviate the underlying oxidative burden, or its high cellular permeability may allow it to regulate gene expression pathways involved in antioxidant defense [2, 6]. Consequently, our findings demonstrate that HRW plays a vital role in preserving membrane structure and maintaining cellular integrity under NP-induced stress.

### Non-enzymatic antioxidants

#### Thiol compounds

Sulfur metabolism products play a crucial role in HM detoxification [69]. In this study, ZnO-NP exposure triggered a significant increase in Cyst and GSH production in arugula seedlings, mirroring previous observations in pomegranate callus [58]. In contrast, ZnO-NP treatment reduced leaf Cyst and GSH concentrations, suggesting that mature foliar tissues may rely on alternative mechanisms to manage ROS. HRW treatment significantly enhanced Cyst and GSH accumulation in ZnO-NP-stressed plants, helping to boost their capacity to resist oxidative stress. This supports the idea that HRW can promote sulfur-containing antioxidants to alleviate (a)biotic stress. These results are consistent with Cui et al. [52], who reported that HRW increased GSH synthesis in Hg-stressed alfalfa seedlings, reduced ROS levels, and reversed oxidative damage. While Cyst is recognized for its role in regulating excess ROS during environmental stress, data regarding HRW’s specific modulation of plant Cyst concentrations has not been previously reported. Our findings thus provide novel evidence that HRW actively stimulates sulfur metabolic pathways, specifically enhancing both Cyst and GSH levels, to reinforce antioxidant defenses under NP-induced toxicity.

#### Ascorbic acid

Ascorbic acid is a foundational antioxidant required to neutralize ROS and maintain metabolic homeostasis under environmental stress [70–73]. In this study, ZnO-NP exposure significantly elevated AsA concentrations in both arugula seedlings and leaves, confirming its active recruitment against NP toxicity [57]. However, this autonomous up-regulation proved insufficient to fully repair the sustained oxidative damage. Conversely, HRW treatment significantly lowered these stress-induced AsA levels, bringing them closer to baseline controls. This reduction suggests a lower baseline oxidative burden within the plant, likely driven by two complementary mechanisms: decreased ZnO-NP uptake and accumulation, alongside a more efficient AsA-GSH cycle for H_2_O_2_ clearance [51]. This is strongly supported by Lin et al. [74], who demonstrated that molecular hydrogen maintains redox homeostasis via the transcriptional upregulation of key AsA-GSH cycle enzymes. Rather than relying on the overproduction of individual antioxidants, HRW appears to optimize the efficiency of existing cyclic antioxidant pathways to neutralize stress. These results raise intriguing questions that deserve further exploration.

#### Total phenolic compounds

Phenolics, a major class of plant secondary metabolites, crucial for scavenging excess ROS under stress, significantly increased in arugula seedlings exposed to ZnO-NPs, both with and without HRW. This accumulation suggests that ZnO-NPs trigger oxidative stress, activating cellular defense mechanisms, a finding aligned with Radi et al. [57], who noted phenolic-mediated ROS scavenging in ZnO-NP-treated calli. Conversely, ZnO-NPs alone significantly reduced phenolic concentrations in leaves, implying a localized reliance on non-phenolic antioxidant pathways. While HRW alone did not alter baseline phenolic levels, its application with ZnO-NPs differentially modulated phenolic concentrations in both seedlings and leaves. This interaction suggests that HRW mitigates ZnO-NP toxicity by stimulating phenolic biosynthesis and reinforcing defense mechanisms, consistent with Su et al. [8], who observed HRW-induced phenolic stimulation in radish sprouts under UV-A stress. Ultimately, HRW-regulated plants maintain superior redox homeostasis by upregulating ROS-scavenging genes and accumulating phenolic compounds [74]. The data reveal a complex interactive effect between ZnO-NPs and HRW on phenolic biosynthetic pathways, a mechanism requiring further molecular validation.

#### Amino acids

Proline, an important osmoprotectant and antioxidant, helps protect plant cells from HM toxicity by forming non-toxic complexes [75]. In this study, ZnO-NP exposure significantly elevated proline concentrations in both seedlings and leaves, likely as a protective response to NP-induced toxicity and subsequent growth inhibition. This accumulation aligns with Radi et al. [57], who reported increased proline levels in pomegranate calli under ZnO-NP stress. Conversely, HRW treatments suppressed this proline accumulation, resulting in intermediate concentrations compared to control and ZnO-NP-only groups. This reduction suggests that HRW mitigates ZnO-NP-induced oxidative stress, thereby lessening the plant’s reliance on proline for ROS defense. This observation partially aligns with Su et al. [76], who found that HRW enhanced proline production in drought-stressed alfalfa by upregulating Δ1-pyrroline-5-carboxylate synthetase activity and downregulating proline dehydrogenase activity. This dual capacity suggests that HRW’s modulation of proline biosynthesis is stress- and context-dependent.

Amino acids mitigate HM toxicity in plants primarily via chelation [77]. In the present study, ZnO-NPs stimulated amino acid synthesis in both seedlings and leaves, suggesting a direct link to enhanced NP tolerance. This aligns with Abdel-Wahab et al. [78], who reported that amino acids are essential for significant ZnO-NP uptake in *Solanum nigrum*. The elevated leaf proline concentrations point to a potential role for this amino acid in chelating ZnO-NPs in the foliage. The potential interactions between HRW and amino acid metabolism warrant further investigation.

#### Soluble proteins

As versatile biomolecules, proteins govern essential cellular functions, including catalysis, transport, and structural support [79]. Here, ZnO-NP treatment significantly increased soluble protein concentrations in seedlings but markedly decreased them in leaves. HRW exposure, with or without ZnO-NPs, had no notable effect on soluble protein levels in either tissue. The observed increase in soluble proteins in seedlings likely contributes to enhanced cellular defense against oxidative stress induced by ZnO-NPs [80]. However, the direct influence of HRW on soluble protein metabolism requires further investigation.

### Enzymatic antioxidants

#### Superoxide dismutase

ZnO-NP exposure induced oxidative stress and ROS production in arugula seedlings and leaves. It was, therefore, imperative to elucidate the role of antioxidant enzymes in ROS detoxification. SOD converts these ROS to H_2_O_2_, which CAT and POD then decompose [81]. Exposure to ZnO-NP markedly decreased SOD activity in arugula seedlings and leaves, likely due to enzyme depletion during heavy ROS scavenging. This finding is consistent with previous reports showing reduced SOD activity in wheat and tobacco cells under ZnO-NP stress [28, 82]. The HRW application enhanced SOD activity in ZnO-NP-treated seedlings and leaves. HRW is known to boost antioxidant capacity and improve plant tolerance to various abiotic stresses, including HMs, salinity, and oxidative damage [2, 3, 6, 82]. Notably, this HRW-induced SOD upregulation was more pronounced in seedlings than in leaves.

#### Catalase

ZnO-NP treatment reduced CAT activity in both arugula seedlings and leaves, likely due to severe oxidative stress and direct enzyme inhibition. Farghaly et al. [28] reported that ZnO-NPs exert mixed inhibition by binding to both the enzyme and the enzyme-substrate complex. HRW treatments enhanced NP tolerance by restoring and elevating CAT activity. This suggests that HRW can enhance CAT activity, thereby improving the plants’ ability to tolerate ZnO-NP toxicity. According to these results, upregulation of antioxidant enzymes has been noted in alfalfa [2], rice [72], and arabidopsis [3]. Mechanistically, the rapid membrane diffusion of H_2_ can stimulate the transcription of antioxidant genes (such as SOD, APX, POD, and CAT) [7]. Furthermore, H₂ can directly reduce ROS in vivo [1] and quench H₂O₂, but it fails to neutralize singlet oxygen [3].

#### Peroxidase

In line with SOD and CAT trends, ZnO-NP stress significantly enhanced POD activity in arugula seedlings and leaves. This elevation serves as an adaptive marker for metallic toxicity and oxidative stress [28], as previously documented in *Solanum nigrum* cells under ZnO-NP exposure [78]. Conversely, the application of HRW and ZnO-NPs decreased POD activity. However, applying HRW to ZnO-NP reduced POD activity, likely by decreasing H_2_O_2_ levels. Nevertheless, HRW effectively alleviated ZnO-NP-induced oxidative damage, as evidenced by lower MDA content. While HRW can upregulate antioxidant genes like POD and APX at the transcriptional level [52, 83], its primary protective role relies on the unique selectivity of molecular H_2_. Unlike traditional antioxidants, H_2_ selectively neutralizes highly cytotoxic oxygen radicals without disrupting the beneficial ROS molecules essential for cellular signaling pathways [5]. This selectivity preserves membrane stability and cellular viability during NP stress.

## Conclusion

The use of ZnO-NPs is increasing, but research shows they can negatively affect plant growth and physiological processes. Deploying defense mechanisms is vital for plant survival, but this process commonly hinders plant growth. Elucidating the molecular processes by which plants balance development and defense can significantly enhance plant breeding and engineering techniques for selecting superior genetic features that optimize plant fitness. We investigated the sequential effect of presoaking arugula seeds in HRW and then applying ZnO-NPs at two growth stages: 7-day-old seedlings and one-month-old plants. The soaking period may have primed the plant, leading to physiological changes that strengthened its oxidative defense system against the later application of ZnO-NPs. This study revealed that exposure to ZnO-NPs resulted in lower plant growth, reduced concentrations of photosynthetic pigments, diminished membrane stability, and decreased levels of SOD and CAT. Treatment of arugula plants with ZnO-NPs led to a substantial accumulation of ROS, which was reflected in significantly elevated MDA levels. We observed that HRW appears to lessen ZnO-NP stress in plants, but the degree to which it offers protection differs across various plant developmental stages. From a practical perspective, HRW seed-priming represents a cost-effective, scalable strategy to protect crops against the growing threat of industrial nanoparticle soil contamination. Furthermore, optimizing the timing of HRW applications based on crop growth stages could maximize yield protection while reducing dependency on chemical inputs. Despite our findings suggesting that HRW could alleviate ZnO-NP stress, more studies are essential to validate its effectiveness and clarify the mechanisms involved. Although direct transcriptomic or proteomic evidence under NP stress conditions remains limited, the consistency of HRW-mediated responses across different abiotic stresses strongly suggests a conserved protective role. This approach holds significant value for regions facing emerging nanoparticle contamination in soil or irrigation water, as it promotes sustainable agriculture by bolstering plant resilience without relying on additional chemical protectants. Future studies employing transcriptomic, proteomic, and metabolomic analyses under ZnO-NP exposure are warranted to elucidate the precise molecular targets and signaling pathways involved in HRW-mediated NP stress mitigation.

## Data Availability

Data will be made available on request.

## Abbreviations

AsA: Ascorbic acid
CAT: Catalase
Cyst: Cysteine
DW: Dry weight
EC: Electrical conductivity
η²: Eta squared
EDTA: Ethylenediaminetetraacetic acid
FW: Fresh weight
GSH: Glutathione
H2: Hydrogen
H_2_O_2_: Hydrogen peroxide
HRW: Hydrogen-rich water
OH: Hydroxyl ion
MDA: Malondialdehyde
NPs: Nanoparticles
O_2_: Oxygen
POD: Peroxidase
K: Potassium
PPb: Potassium phosphate buffer
ROS: Reactive oxygen species
SD: Standard deviation
SOD: Superoxide dismutase
TBA: Thiobarbituric acid
DTNB: 5,5'-dithiobis-2-nitrobenzoic acid
TI: Tolerance index
TCA: Trichloroacetic acid
UV: Ultraviolet
Zn: Zinc
ZnO-NPs: Zinc oxide nanoparticles
